# Ligand-Induced Protein Mobility in Complexes of Carbonic Anhydrase II and Benzenesulfonamides with Oligoglycine Chains

**DOI:** 10.1371/journal.pone.0057629

**Published:** 2013-03-05

**Authors:** Vijay M. Krishnamurthy, Venkata S. Raman, Richard A. Mowery, Michelle Hentz, James D. Baleja, Bryan F. Shaw, Krishna Kumar

**Affiliations:** 1 Department of Chemistry, Tufts University, Medford, Massachusetts, United States of America; 2 Department of Chemistry and Biochemistry, Baylor University, Waco, Texas, United States of America; 3 Department of Biochemistry, Tufts University School of Medicine, Boston, Massachusetts, United States of America; 4 Cancer Center, Tufts Medical Center, Boston, Massachusetts, United States of America; University of South Florida College of Medicine, United States of America

## Abstract

This paper describes a biophysical investigation of residual mobility in complexes of bovine carbonic anhydrase II (BCA) and *para-*substituted benzenesulfonamide ligands with chains of 1–5 glycine subunits, and explains the previously observed increase in entropy of binding with chain length. The reported results represent the first experimental demonstration that BCA is not the rigid, static globulin that has been typically assumed, but experiences structural fluctuations upon binding ligands. NMR studies with ^15^N-labeled ligands demonstrated that the first glycine subunit of the chain binds without stabilization or destabilization by the more distal subunits, and suggested that the other glycine subunits of the chain behave similarly. These data suggest that a model based on ligand mobility in the complex cannot explain the thermodynamic data. Hydrogen/deuterium exchange studies provided a global estimate of protein mobility and revealed that the number of exchanged hydrogens of BCA was higher when the protein was bound to a ligand with five glycine subunits than when bound to a ligand with only one subunit, and suggested a trend of increasing number of exchanged hydrogens with increasing chain length of the BCA-bound ligand, across the series. These data support the idea that the glycine chain destabilizes the structure of BCA in a length-dependent manner, causing an increase in BCA mobility. This study highlights the need to consider ligand-induced mobility of even “static” proteins in studies of protein-ligand binding, including rational ligand design approaches.

## Introduction

Understanding the driving forces for the non-covalent association of a protein with a small-molecule ligand–e.g., interactions based on electrostatics, hydrophobicity, and solvation–is an area of high interest for chemistry, biology, and medicine. Achieving a deep understanding of these underlying forces would enable the realization of the holy grail of “rational ligand design”: the design of a high-affinity ligand for a protein solely from its three-dimensional structure and/or genetic sequence. While this achievement would significantly advance fundamental molecular science and thus be intellectually satisfying, it would also suggest ways to reduce the enormous and rising R&D cost for the discovery of novel therapeutics, currently estimated at more than $1 billion per drug [1].

While significant progress has been made in understanding non-covalent association, a number of challenges still remain [2]–[5]. Two of these challenges are: (i) developing an understanding of the role of water in the binding process that goes beyond the low level of resolution offered by many theories of the so-called “hydrophobic effect” [6]–[10], and (ii) quantifying the conformational mobility for the macromolecule and the ligand in the complex [3], [11]–[13]. Both solvation and mobility are believed to manifest themselves primarily, albeit not exclusively, in the entropy of binding. Our inability to predict accurately, or to rationalize quantitatively, entropy has inhibited our ability to solve the aforementioned challenges. The challenge is exacerbated as assessments of entropy, in general, cannot be derived solely from “static” structures of protein/ligand complexes available from NMR or X-ray methods [13], [14]. Moreover, the concept of enthalpy/entropy compensation [3], [15]–[17]–the positive correlation of changes in enthalpy and entropy as one variable of the system is changed (often perturbations to ligand structure) that serves to minimize the net effect on the free energy (affinity)–has been described in many situations and further serves to complicate efforts focused on rational ligand design. To address these challenges, careful studies of the separate influence of enthalpy and entropy of binding of systematically varied ligands to well-characterized proteins are necessary to develop useful guiding principles with potential applicability to rational ligand design.

**Figure 1:**
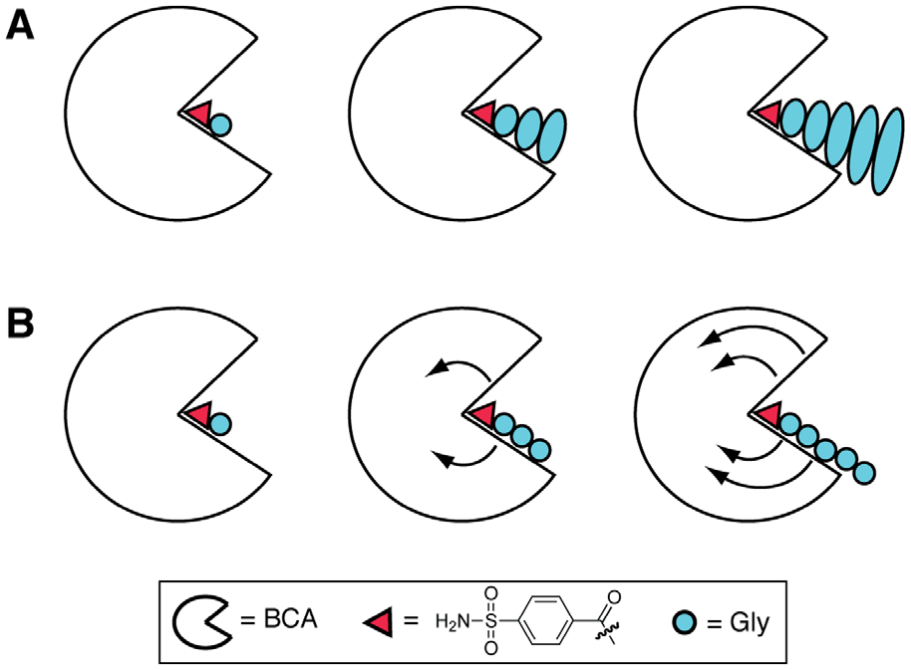
Schematics for potential models for binding of benzenesulfonamide ligands with glycine chains to bovine carbonic anhydrase II (BCA). Complexes of BCA with ligands with one, three, or five glycine subunits are shown. (**A**) “Ligand mobility” model in which binding of Gly subunits farther from the benzene ring (distal subunits) destabilize the binding of subunits closer to the ring (proximal subunits). The sizes of the ellipses for the subunits are roughly proportional to the mobility of the subunits. (**B**) “Protein mobility” model in which the binding of the Gly chain destabilizes interactions within BCA itself. The curved arrows denote mobility, which increases with increasing chain length of the ligand.

We previously conducted one such study, using isothermal titration calorimetry to characterize the binding of a model protein, bovine carbonic anhydrase II (BCA), to benzenesulfonamide ligands with *para* substituents of oligoglycine, oligo(ethylene glycol), and oligosarcosine chains with increasing lengths of 1 to 5 subunits [18]. X-ray crystallographic studies revealed that these ligands bind in a conserved orientation with BCA [19], [20]: the sulfonamide anion binds to the Zn^2+^ cofactor of BCA, the benzene ring interacts with hydrophobic patch of BCA, and the chain of the ligand interacts with a hydrophobic wall along the conical cleft of the enzyme [21]. This near constant orientation allowed analysis of thermodynamic data without complications from different modes of binding. Our results demonstrated that for all three series of ligands, as chain length increased from 1 to 5 subunits, the enthalpy of binding became less favorable by 1–2 kcal mol^−1^ and the entropy became less unfavorable by a compensating amount. In addition, the change in heat capacity–the so-called “signpost” of the hydrophobic effect [22]–[25]–did not vary across the series. These data were consistent with two models for mobility at the protein-ligand interface (“interfacial mobility”): one based on increasing *ligand* mobility, in which subunits farther from the benzene ring (“distal” subunits) actively destabilize the binding of ones closer (“proximal” subunits), and the other based on increasing *protein* mobility [3], in which the chain of the ligand induces a “loosening” of the internal structure of BCA (Figure 1). With no data to support the presence of protein mobility, and with the prevailing view of BCA as a remarkably static enzyme with or without bound ligand [3], [21], we postulated that the “ligand mobility” model (Figure 1A) was the most likely one to explain the data.

Recently Homans and co-workers examined the mobility of benzenesulfonamide ligands with Gly chains in complex with BCA, estimating ligand mobility by measuring NMR relaxation parameters and protein mobility using molecular dynamics simulations [26]. They proposed that the binding of Gly subunits was in fact mutually reinforcing, i.e., the binding of distal Gly subunits stabilized the binding of more proximal ones, and that the observed increasingly less unfavorable entropy (decrease in -TΔS) with chain length was due to increased mobility of residue side chains across BCA, mostly within the active site itself. While this study contributes much to our understanding of this system, it did not measure or estimate three important properties in the CA/ligand interaction: (i) the mobilities of the *free* ligands in solution, which can serve as a baseline to which to compare the mobilities in the protein-ligand complex, (ii) the mobilities of specific subunits within the ligand when complexed with BCA, as the ligand’s chain length was increased; these data would gauge the degree of reinforcement between subunits, and (iii) an *experimental* assessment of protein mobility.

Here we report the results of a biophysical study that addresses the three aforementioned issues. We used NMR spectroscopy to evaluate the mobility of the ligand at one specific position–the subunit nearest to the benzene ring–with increasing chain length, both free in solution and in complex with BCA. We experimentally evaluated mobility of the protein using hydrogen/deuterium exchange and mass spectrometry to evaluate the accessibility of backbone amides of BCA in the presence of ligands with Gly chains of increasing length. Contrasting with the conclusions of Homans and co-workers, our NMR data demonstrate that, in fact, the binding of the first subunit is *not* affected by the binding of more distal subunits (within experimental error), and implies that the other subunits behave similarly, with each subunit binding without stabilization or destabilization by the binding of more distal subunits. These data refute the ligand mobility model (Figure 1A). The hydrogen/deuterium exchange studies suggest that the effect of the oligoglycine chain on BCA mobility is the origin of the thermodynamic profile of these ligands, with less unfavorable entropy resulting from the increased protein mobility and the less favorable enthalpy from fewer ordered internal hydrogen bonds; the net effect being enthalpy/entropy compensation across the series. Thus, these data support the “protein mobility” model (Figure 1B). To the best of our knowledge, our results represent the first experimental demonstration that BCA is not the rigid, static globulin that has been typically assumed [21]. but that BCA experiences small structural fluctuations upon binding of ligands (and possibly during normal biological catalysis). These small fluctuations could be the source of past peculiarities in thermodynamic measurements of the binding of ligands to BCA.

## Results and Discussion

### Synthesis of Ligands

**Figure 2:**
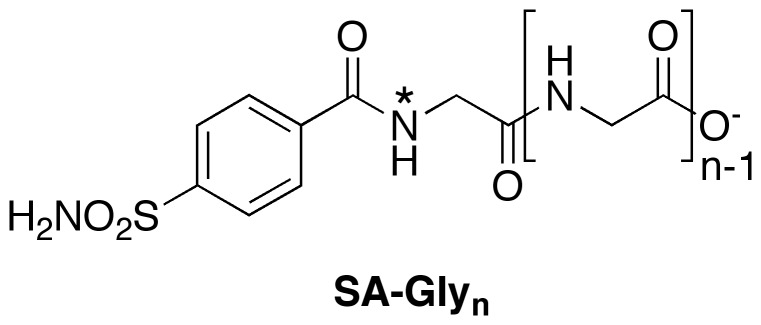
Structure of benzenesulfonamide ligands with Gly chains (SA-Gly_n_). The ligands used in the reported studies varied in length from 1 to 5 subunits (n = 1–5). The ^15^N label at the first Gly subunit is denoted by an ‘*’.

The initial goal of this study was to test the “ligand mobility” model (Figure 1A) [18]. In this model, the binding of distal Gly subunits destabilizes the binding of more proximal ones. We rationalized that the simplest test of this hypothesis would involve examining the change in mobility of the Gly subunit closest to the benzene ring (the “first” subunit), when the ligand was in complex with BCA, as the chain was lengthened. To enable these studies, we used conventional solid-phase methods to synthesize a series of benzenesulfonamide ligands (SA-Gly_n_, n = 1 to 5; Figure 2) with Gly chains of varying length and with a constant first subunit of an ^15^N-labeled Gly (depicted as “*”; see Experimental Section).

### Distal Subunits of SA-Gly_n_ Ligands Reduce Mobility of First Subunit when Ligand is Free in Solution

Combining NMR spectroscopy with ^15^N-labeled amino acid residues is a common method to measuring the mobility (dynamics) of specific residues within peptides and proteins [27]. For our purposes, measuring ^15^N NMR chemical shifts and relaxation parameters of the first subunit of the SA-Gly_n_ ligands allowed us to determine the variation of the chemical environment and mobility of this subunit as the length of the chain (n) increased.

**Figure 3:**
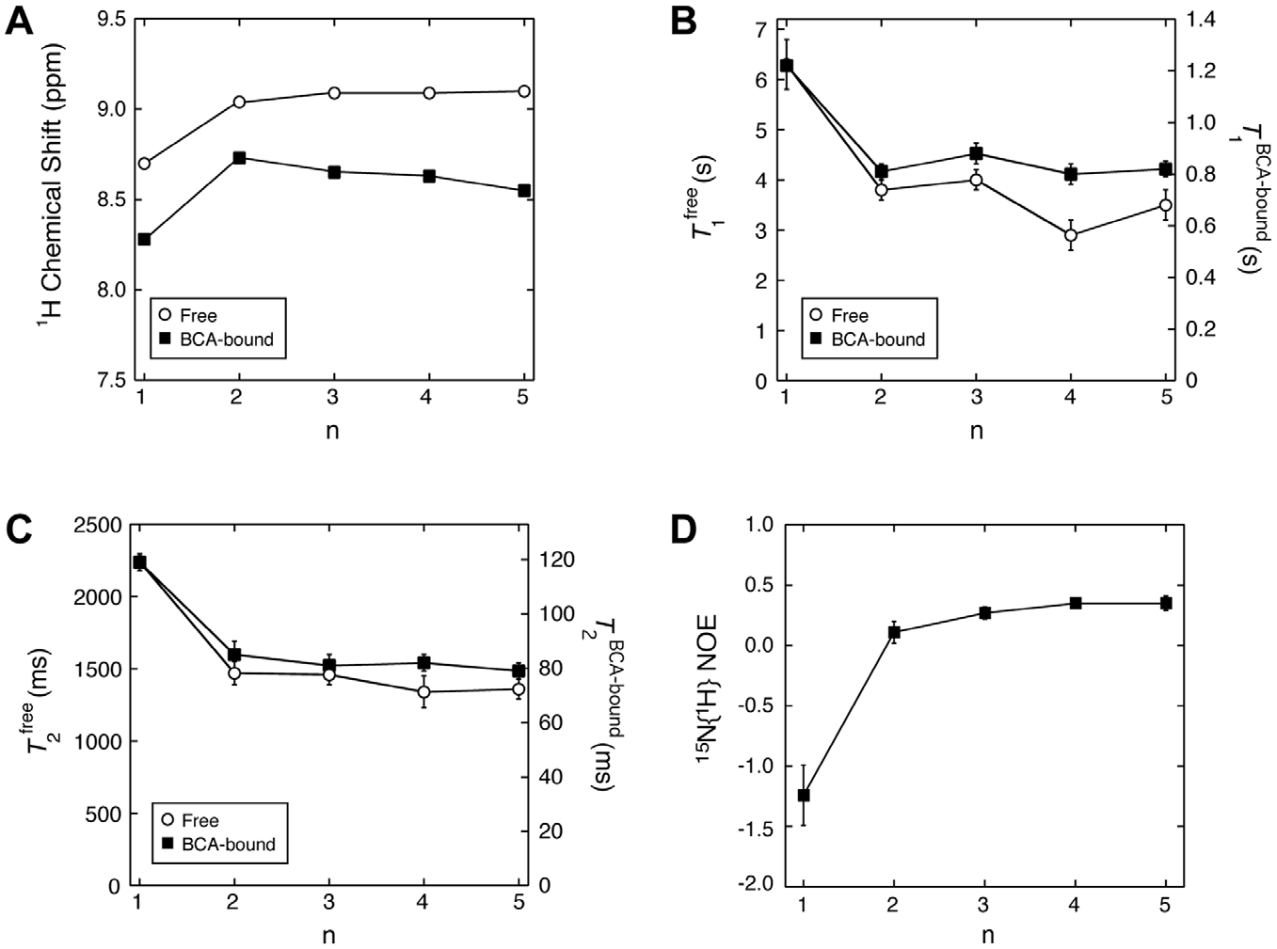
NMR data for the amide closest to the benzene ring (the “first” subunit) for benzenesulfonamide ligands with Gly chains (SA-Gly_n_). The variation of these NMR data with chain length (n) of the ligand are compared for the case when the ligands are free in buffered solution (open circles) and when they are in complex with bovine carbonic anhydrase II (BCA; closed squares). (A) Chemical shifts for the “first” amide proton exhibit a nearly constant difference between free and BCA-bound forms across the ligand series. ^15^N NMR relaxation times of *T*_1_ (B) and *T*_2_ (C) across the SA-Gly_n_ ligand series exhibit a nearly constant relative decrease from the free to the BCA-bound forms (revealed by separate axes for free and for BCA-bound data). (D) Values of ^15^N-^1^H steady-state NOE for the SA-Gly_n_ ligands when bound to BCA suggest a different dynamic environment for the SA-Gly_1_ ligand relative to the other, longer ligands (n≥2), for which the trend shows little dependence on chain length (n).

Examining ^15^N NMR parameters of the *free* ligand establishes a reference state to which the values for the ligand in complex with BCA can be compared in order to infer changes in mobility that occur upon binding. The NMR chemical shift is a sensitive probe of the chemical environment around a magnetic nucleus. We measured chemical shifts for the first subunit when the ligand was free in an aqueous phosphate buffer by ^1^H NMR spectroscopy. We conducted these studies at a value of pH (6.8) slightly lower than that used for the previous ITC studies (7.5; [18]) in order to facilitate visualization of the resonances of the amide protons by slowing down their hydroxide-catalyzed exchange (and without shifting the equilibrium too drastically to the carboxylic acid form of the ligand from the carboxylate form) [28]. The chemical shift for the first subunit was significantly different in SA-Gly_1_ than in the ligands with longer chains, SA-Gly_n_ n = 2–5 (Δ*δ = *0.3–0.4 ppm; Figure 3A). Further, the chemical shift did not vary across the series when n ≥2 (Δ*δ* <0.1 ppm). These observations support the following conclusions: (i) the first subunit is in a different chemical environment when it is the only subunit in the chain (i.e., in SA-Gly_1_) than when at least one subunit is more distal to it (i.e., SA-Gly_n_ n ≥2), and (ii) the chemical environment of the first subunit is invariant after the addition of at least one more distal subunit.

Relaxation of the ^15^N nucleus of labeled amino acid residues is dominated by the directly attached amide proton, and so ^15^N NMR relaxation parameters can be directly attributed to the dynamics of the amide bond vector itself, free of external complications [29]. We measured the ^15^N NMR relaxation times *T*_1_ and *T*_2_ using ^15^N-^1^H HSQC spectra and established pulse sequences (see Materials and Methods). In line with the chemical shift data, relaxation parameters of the first subunit show little variation with chain length when there are at least two subunits in the chain, SA-Gly_n_ with n ≥2 (coefficients of variation of 13% and 9% for *T*_1_ and *T*_2_, respectively), but these parameters differ significantly when there is only one subunit in the chain, SA-Gly_1_ (decrease in *T*_1_ of 40–50% relative to SA-Gly_1_, and in *T*_2_ of 30–40%). From these data, we infer that the first subunit has greater mobility (dynamics) in SA-Gly_1_ than in the longer ligands, and that the first subunit is stabilized by the addition of at least one distal subunit. This result is not unexpected, given that amino acid residues at the termini of peptides generally have greater mobility than internal residues.

### A Similar Trend in Relaxation Parameters for the First Subunit of SA-Gly_n_ Ligands in Complex with BCA and Free in Solution Suggests that it Binds to BCA without Stabilization or Destabilization by Other Subunits

With the baseline of dynamics of the free ligands established, we moved to characterizing the dynamics of the ligands in complex with BCA. The difference in chemical shift of the ligand between the free and BCA-bound states reflects the change in chemical environment of the ^15^N nucleus upon binding (i.e., transfer from solution to the active site of BCA). We determined chemical shifts of the first subunit in the SA-Gly_n_ ligands in complex with BCA using ^1^H-^15^N HSQC spectra (see Experimental Section). The trend in chemical shift with chain length was the same when the ligand was in complex with BCA or when it was free. Both cases showed a significant shift from SA-Gly_1_ to SA-Gly_2_ and then little variation through SA-Gly_5_ (Figure 3A). These similar trends resulted in a virtually constant difference in chemical shifts of the first subunit between BCA-bound and free states across the ligand series (Δδ = 0.4±0.1 ppm; y-distance between the lines in Figure 3A). This observation suggests that the difference in chemical environment between the free and BCA-bound states is constant across the series, i.e., that the first subunit undergoes a similar change in chemical environment moving from aqueous solution to the active site of BCA regardless of whether or not there are more distal subunits.

We measured ^15^N NMR relaxation parameters (*T*_1_ and *T*_2_) of the first subunit in the SA-Gly_n_ ligands in complex with BCA (using the same methods as for the free ligands in the previous section; see Materials and Methods). Our measured values of *T*_1_ and *T*_2_ for SA-Gly_1_ in complex with BCA show exactly the same trends as previously reported, except with differences in magnitude (by ∼5% and 30%, respectively, discussed in the next section) [26]. Values of *T*_1_ (Figure 3B) and *T*_2_ (Figure 3C) of the first subunit in SA-Gly_1_ were higher than in the longer ligands (n≥2); this observation is consistent with the data for the free ligands and implies that the first subunit has greater mobility when in SA-Gly_1_ than when in the longer ligands, both in the complex of BCA and free in solution. Further, the relaxation parameters did not vary with chain length (outside of the uncertainties) when there were at least two Gly subunits in the chain (SA-Gly_n_ with n ≥2), which suggests that, on the time scales sensitive to the ^15^N relaxation experiments, the mobility of the first Gly subunit when bound to BCA is the same regardless of the chain length of the ligand when there is at least one subunit distal to it. Analyzing the variation of the relaxation parameters with chain length (n) quantitatively reveals a similar trend for the free ligand and BCA-bound cases: *T*_1_ and *T*_2_ for the first subunit decrease by the same relative amount from n = 1 to n ≥2 for both cases (Figure 3B and 3C; the free and BCA-bound cases have been plotted on different maximum scales of the y-axes to show this relative trend). This observation suggests that the relative decrease in mobility for the first subunit when moving from free solution to the active site of BCA is the same regardless of chain length and whether or not there are subunits distal to it. The implication of these data is that the binding of the first subunit is not influenced (either positively or negatively) by the binding of the other subunits, and does not involve a destabilization of, or by, other subunits as postulated in the “ligand mobility” model (Figure 1A; see next section).

**Figure 4:**
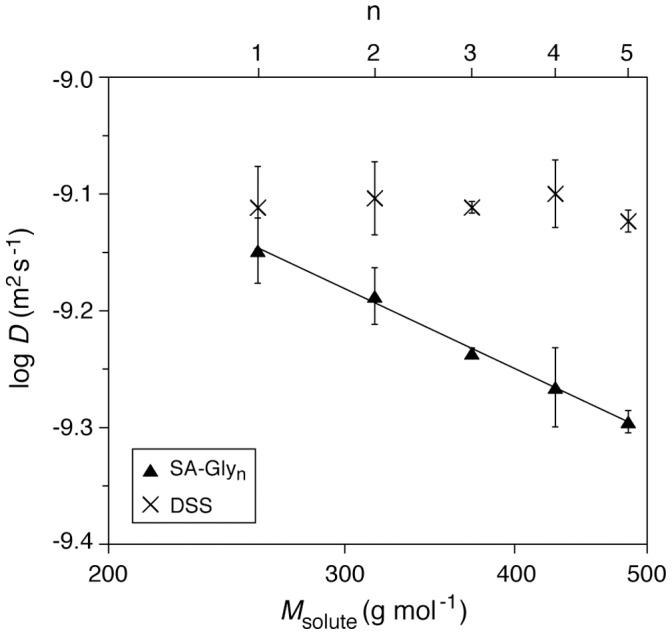
Variation of diffusion coefficient (*D*) with molecular weight (*M*_solute_) and chain length (n) of SA-Gly_n_ ligands. The constant values of *D* measured for the internal standard DSS (4,4-dimethyl-4-silapentane-1-sulfonic acid) in the different samples demonstrate the consistency of experimental conditions (e.g., viscosity). Error bars represent standard deviations calculated from the full-width at half maximum of the peaks in DOSY spectra. A linear fit to the SA-Gly_n_ data (log *D* vs log *M*_solute_) is shown with fitting parameters of −0.54±0.02 (slope) and −7.83±0.05 (y-intercept), yielding an R^2^ of 0.996.

Steady-state ^15^N{^1^H} NOE data are also diagnostic of the mobility of subunits within peptides and proteins, ranging from −3.6 for fast motions to +0.82 for slow motions [30]. We used established pulse sequences to estimate steady-state values of NOE for the first subunit in the SA-Gly_n_ series of ligands. Consistent with the *T*_1_ and *T*_2_ data, the NOE data reveal higher mobility of the first subunit in SA-Gly_1_ (and fast motions in general) than in the other, longer ligands (with much slower motions), and that the NOE data are essentially invariant when there is at least one distal subunit, n ≥2 (Figure 4D). We discuss these data more quantitatively in the next section.

### Model-free Analysis of NMR Relaxation Data Quantitatively Confirms Trends in Parameters

The model-free formalism, described by Lipari and Szabo [31], [32] and extended by Clore et al. [33], has been routinely applied to fit protein NMR data (*T*_1_, *T*_2_, and steady-state NOE) to estimate amplitudes and time-scales of intramolecular motions [27]. The extended model-free formalism consists of the following parameters: *S* ^2^ (which describes the overall degree of spatial restriction of the internal motion of the ^1^H-^15^N bond vector, varying from 0 for no restriction to 1 for complete restriction), *S*_f_^2^ and *S*_s_^2^ (which take into account spatial restrictions of internal motions occurring at time scales that vary by at least an order of magnitude; the product of these two terms is equal to *S* ^2^), *τ*_e_ (the effective correlation time of internal motions that differ from that for tumbling of the entire protein), and *R*_ex_ (the rate of chemical or conformational exchange). The relaxation data can be fit to five models [34], which differ in the parameters that are allowed to vary: *S* ^2^ (model 1), *S* ^2^ and *τ*_e_ (model 2), *S* ^2^ and *R*_ex_ (model 3), *S* ^2^, *τ*_e_, and *R*_ex_ (model 4), and *S*_f_^2^, *S*_s_^2^, and *τ*_e_ (model 5). We used the software program Modelfree 4.20 to estimate dynamics quantitatively from the measured relaxation parameters for the SA-Gly_n_ ligands in complex with BCA (see Experimental Section for details) [34], [35].

A number of criteria have been proposed for selecting the most appropriate of the five models for different residues [34], [36], [37]. On the basis of simulated data sets, d’Auvergne and Gooley argued that Akaike’s Information Criterion (AIC) [38] was best suited for model selection [37], and thus we utilized this approach for the SA-Gly_n_ ligands. We observed similar trends in order parameters regardless of the model adopted for the different ligands. Table 1 summarizes the model-free fitting parameters and models for the first subunit in the SA-Gly_n_ ligands. For n = 3, 4, and 5, the data were best fit by model 5 involving internal motions on two different time scales. However, the motion on the fast time-scale scale was severely restricted (*S*_f_^2^>0.9) and near the limit of complete restriction (*S*_f_^2^ = 1). Thus, motion on the fast time-scale makes only a weak contribution to the internal dynamics, which is thus primarily due to motion on only one time-scale. For n = 2, the data were well fit by models 5 and 2; model 2 includes the same parameters as model 5 but with motion on only one time-scale (i.e., *S*_f_^2^ is constrained to unity). The model-free parameters were within error in both models, and model 2 was adopted based on the AIC (Table 1). For n = 1, model 4, which includes the term *R*_ex_ for contributions of chemical (conformational) exchange to transverse relaxation (*T*_2_) in the µs-ms time frame, provided the best fit.

**Table 1:** Extended model-free parameters for the ^15^N-^1^H bond vector of the amide closest to the benzene ring (the “first” subunit) in SA-Gly_n_ ligands in complex with BCA.

n	Model	S^2^	S_f_^2^	S_s_^2^	t_e_ (ps)	R_ex_ (s^−1^)	AIC^a^
1	2^b^	0.49±0.01	1^c^	0.49±0.01	90±5	0^c^	15.7
1	4	0.35±0.04	1^c^	0.35±0.04	123±9	2.3±0.7	6.0
2	2^b^	0.72±0.04	1^c^	0.72±0.04	510±100	0^c^	4.5
2	5	0.69±0.04	0.97±0.04	0.71±0.04	550±110	0^c^	6.0
3	5^b^	0.73±0.04	0.92±0.04	0.79±0.04	490±150	0^c^	6.0
4	5^b^	0.72±0.04	0.95±0.03	0.76±0.03	750±90	0^c^	6.0
5	5^b^	0.75±0.04	0.96±0.03	0.78±0.03	670±120	0^c^	6.0

a Value from Akaike’s Information Criterion (AIC) [38]. The model with the lowest value is the one selected [37].

b Accepted model for the ligand (see text).

c Parameter is held constant in this model.

In order to determine whether contributions to conformational exchange might be contributing to transverse relaxation in SA-Gly_1_, we measured values of *T*_1ρ_ using established pulse sequences (see Experimental Section). Values of *T*_1ρ_ measure transverse relaxation in the rotating frame, and are expected to be identical to values of *T*_2_ if no exchange processes that are slower than the strength of the spin-lock field occur (i.e., *R*_ex_ is not less than 2500 s^−1^) [39], [40]. Values of *T*_1ρ_ and *T*_2_ were similar for all SA-Gly_n_ ligands (differing by <10%; Figure S1), a result that suggests that conformational exchange likely does not contribute to transverse relaxation for any of the ligands and that the fit of model 4 for SA-Gly_1_ (with a small *R*_ex_ contribution of 2 s^−1^) might be a limitation of only having relaxation data at one magnetic field strength. For this reason, we adopted model 2 for SA-Gly_1_, although the trend of the overall order parameter (*S* ^2^) with chain length (n) is the same regardless of the assumed model. This analysis reinforces our previous conclusions from the individual relaxation parameters with a quantitative analysis of ligand dynamics, revealing that the first subunit of the SA-Gly_n_ ligands has significantly greater mobility when it is the only subunit in the chain (n = 1) than when there are distal subunits to it and that the dynamics of the first subunit are essentially the same regardless of the number of such distal subunits present (Table 1). The values of *S* ^2^ for SA-Gly_n_ with n ≥2 are in the range for ordered backbone amides within proteins (0.7–0.9), revealing that the first subunit is tightly bound in the active site of BCA when it is not the most distal subunit.

In work complementary to that described here, Homans and co-workers recently estimated the mobilities of a related series of SA-Gly ligands when bound to BCA [26]. They examined one ligand in common with our studies, SA-Gly_1_ (series 1, residue 1 in their nomenclature), and one which would be an extension of our series, SA-Gly_6_ (series 2, residue 6). If we assume that the dynamics of the first subunit are similar in SA-Gly_6_ and in SA-Gly_5_ (a justifiable assumption given the independence of *S* ^2^ with n when n ≥2; Table 1), then both SA-Gly_1_ and SA-Gly_5_/SA-Gly_6_ are found to be slightly (∼30%) less ordered in their measurements than ours. This difference could be due to the use of different batches of BCA or some other difference in experimental conditions. However, most importantly, the fact that their data reveal a similar trend of *S* ^2^ with chain length (n) provides independent support for this trend being correct.

### NMR Data Suggest that Each Glycine Subunit of SA-Gly_n_ Binds to BCA without Ligand Destabilization, and Likely without Being Influenced by the Binding of More Distal Subunits

Homans and co-workers studied two series of benzenesulfonamide ligands with oligoglycine chains: series 1 comprising six ligands of varying chain length (n = 1–6) in which the most distal, carboxy-terminal subunit was ^15^N-labeled, and series 2 comprising six ligands of constant chain length (n = 6) with the position of the ^15^N label varied at the six possible amides [26]. For a label at a given position, the authors observed that values of *S* ^2^ were lower for the series 1 ligand than for the analogous series 2 ligand, and from these data concluded that the addition of distal subunits *reduced* the dynamics of more proximal subunits. The authors did not, however, examine the relaxation parameters of the free ligands in the absence of BCA.

Our NMR results are consistent with their published report as we observe a decrease in dynamics of the first subunit of the SA-Gly_n_ ligands when distal subunits are present (Table 1). Moreover, our results reveal that the dynamics of the first subunit are constant regardless of the number of distal subunits present (i.e., *S* ^2^ does not vary with n when n ≥2; Table 1), and that the quantitative change in dynamics of the first subunit with chain length is the same whether the ligand is free or bound to BCA (Figure 3). From these observations, we conclude that the first subunit binds to BCA with neither stabilization nor destabilization by the binding of other subunits. On the simplifying basis of Occam’s razor, we speculate that the dynamics of the other positions will behave similarly as for the first subunit, and that the greater dynamics of a given labeled position in the series 1 than the series 2 ligands is a manifestation of the greater intrinsic dynamics of carboxy terminal residues than internal residues, and would be reflected in increased dynamics of the series 1 than series 2 ligands when free in solution. We, thus, believe that the binding of each subunit of the SA-Gly_n_ ligands is in fact not influenced by the binding of more distal subunits (i.e., without positive reinforcement or destabilization), a finding that contrasts with the conclusions of Homans and co-workers.

Taken together with the work of Homans and co-workers, our data reveal that the binding of the subunits is not destabilizing as previously hypothesized (Figure 1A) and that there must be another origin of the less unfavorable entropy with chain length observed from calorimetry [18].

### Aggregation of Ligands Cannot Explain Trend in Thermodynamics

Recent work has suggested that drug-like molecules can aggregate in aqueous solution and that this aggregation manifests itself as artifactual binding in assays [41], [42]. While we had no evidence for aggregation for the SA-Gly_n_ ligands, the solubility of oligoglycine peptides (molecules similar to SA-Gly_n_ but lacking the benzenesulfonamide moiety) has been shown to decrease precipitously with chain length, from >200 mg mL^−1^ (2.8 M) for glycine to ∼0.6 mg mL^−1^ (1.6 mM) for hexaglycine [43]. The Gly peptides could, in principle, aggregate into small multimers or other structures at concentrations below their solubility limits. There was, thus, the possibility that the SA-Gly ligands used here might aggregate into multimers at the concentrations used for the ITC studies (∼0.2 mM), and that their propensity to do so would scale with the length of the chain. In this “aggregate” model, aggregation would be alleviated upon binding by BCA resulting in a favorable contribution to entropy that scaled with the length of the chain.

Translational diffusion coefficients (*D*) of the free SA-Gly_n_ ligands provide a test of this aggregation model, as *D* would be expected to be lower for a multimer than for a monomer. The Wilke-Chang correlation (eq 1) relates *D* with molar volume by a semi-empirical modification of the more general Stokes-Einstein relation, and has been applied successfully to small molecules in low molecular-weight solvents [44], [45]:
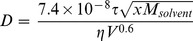
(1)where *D* is the diffusion coefficient (in cm^2^ s^−1^), *T* is the absolute temperature (in K), *x* is an empirical parameter used to quantify the degree of association of the solvent (*x = *1 for a nonassociated solvent, and *x = *2.26 for water) [45], *M_solvent_* is the molecular weight of the solvent (18.0 g mol^−1^ for water) *η* is the viscosity of the solvent (8.899 centipoise, cg cm^−1^ s^−1^, for water at 298 K and 1 atm) and *V* is the molar volume of the solute in (cm^3^ mol^−1^). If we make the assumption that the densities (*ρ* in g cm^−3^) of the ligands are the same within error, equation 1 becomes equation 2, which relates *D* of the solute to its molecular weight (*M_solute_* in g mol^−1^):



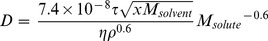
(2)If there were no aggregation of the ligand, *D* should decrease modestly with molecular weight (*M*_solute_): a plot of the logarithms of the two parameters would have a slope of −0.6. However, if there were aggregation, we would expect such a plot to have a more negative slope than −0.6.

Diffusion Ordered SpectroscopY (DOSY) allows the determination of diffusion coefficients of mixtures by using pulse-field gradients in NMR spectroscopy [46]–[48]. A DOSY experiment commonly consists of collecting a series of NMR spectra at varying strengths of the gradient, and then fitting the exponential decay in peak heights to estimate values of *D* for each peak. We collected ^1^H NMR spectra of the SA-Gly_n_ ligands (0.5 mM) under the same conditions as used for the other NMR experiments (see Experimental Section), and fit the data with a bi-exponential decay function in order to allow for the existence of two species within each peak (that would be in the slow exchange on the NMR time scale). The DOSY spectra of all of the ligands revealed cross-peaks corresponding to the ligand (comprising the aryl, amide, and alpha carbon protons), solvent, buffer components, and the internal standard (Figure S2). Importantly, the DOSY analysis revealed only one value of *D* for each of the ligands, suggesting either a homogeneous species (monomer or aggregate) or an average *D* of multiple species that rapidly equilibrated on the NMR time scale.

Figure 4 shows the DOSY-derived values of *D* plotted against molecular weights of the ligand. The linear fit to the data yields a slope of 0.54±0.02, close to the value of 0.6 expected if the ligand were only present as the monomer. The *y*-intercept (−7.83±0.05) is the same (within error) to the value calculated (−7.80) using eq 2 (with *T* = 300 K and *ρ* = 1 g cm^−3^), and provides additional confirmation that the theory explains the data well. We interpret these results to mean that there is no significant ligand aggregation at concentrations of ligand of 0.5 mM and below because of: (i) the calculation of only a single value of *D* for each ligand, and (ii) the calculated value of *D* closely matching the value expected for a monomer calculated from theory (and not significantly lower as would be expected for either a multimer or the weight-average of a multimer and monomer). These data, thus, reveal that a model based on ligand aggregation is *not* the source of the observed thermodynamics in this system.

### Protein Mobility Mediated by Internal Amide Bond Destabilization can Plausibly Explain the Thermodynamic Data

While we had previously believed that the ligand mobility model (Figure 1A) was the most likely one to explain the trend in thermodynamics for the binding of benzenesulfonamide ligands with oligoglycine chains to BCA, we had also hypothesized that destabilization of BCA by the ligand might be occurring [18]. However, with no clear experimental support for such a model, and in light of the dogma of BCA as a static molecule with or without bound ligand [21], we could not comment further on whether this model might be contributing to the observed thermodynamic profile. On the basis of molecular dynamics simulations of SA-Gly_n_ ligands in complex with BCA, Homans and co-workers concluded that the dynamics of amino acid residues within the binding pocket of BCA, in particular the His residues that coordinate the Zn^2+^ cofactor (which in turn binds to the deprotonated sulfonamide), increased with the chain length of the bound ligand [26]. In addition, their simulations suggested that the Zn^2+^-sulfonamide bond lengthened with increasing chain length, which would potentially be another source of a favorable contribution to entropy.

While these results were provocative, an experimental demonstration of increased dynamics of BCA with chain length would establish the plausibility of the “protein mobility” model, especially in light of the common perception of BCA as a remarkably static protein. In order to study the effect of ligand binding on the solution structure and dynamics of BCA, we used amide hydrogen/deuterium (H/D) exchange to probe the backbone structure of BCA in the presence (and absence) of the SA-Gly_n_ ligands. The rate of H/D exchange is a sensitive indicator of structure, and is dependent on the degree of hydrogen bonding and solvent accessibility of backbone amides (although recent work [49], [50] has demonstrated a role for the electrostatic environment). H/D exchange studies have been routinely used to study the dynamics and structural effects of proteins upon binding ligands [3]. The guiding principle of these studies has been that increased dynamics within the protein, primarily at intramolecular hydrogen bonds between backbone amides, allows for increased access of deuterium oxide (D_2_O), resulting in faster amide H/D exchange in the protein.

Since the assignment of ^1^H-^15^N NMR resonances for BCA has not been made [21], [49], we measured the rate of H/D exchange of BCA with electrospray ionization mass spectrometry (ESI-MS) to gain a global estimate of protein mobility. We conducted H/D exchange studies by pre-incubating BCA with or without an SA-Gly_n_ ligand, diluting the sample into D_2_O for a specified time period, quenching the exchange reaction by lowering the pH, and then measuring deuterium incorporation (mass of BCA) by ESI-MS, in a procedure similar to our previous report (see Experimental Section for details) [49]. Under the acidic conditions of quenching, the His residues of BCA that coordinate the Zn^2+^ cofactor become protonated, which has been demonstrated to release the cofactor and the bound sulfonamide [21], [51]. Thus, the measured masses are for BCA alone and not for the BCA/ligand complexes. We also included the ligand *p*-carboxybenzensulfonamide (**SA-OH**) to control for possible effects of the benzenesulfonamide moiety on protein structure. If an increase in chain length were accompanied by an increase in protein dynamics (i.e., Gly chain-induced destabilization of the protein), we would expect to observe an increase in the number of exchanged hydrogens (an increase in mass of BCA) with the chain length (n) of the incubated ligand.

**Figure 5:**
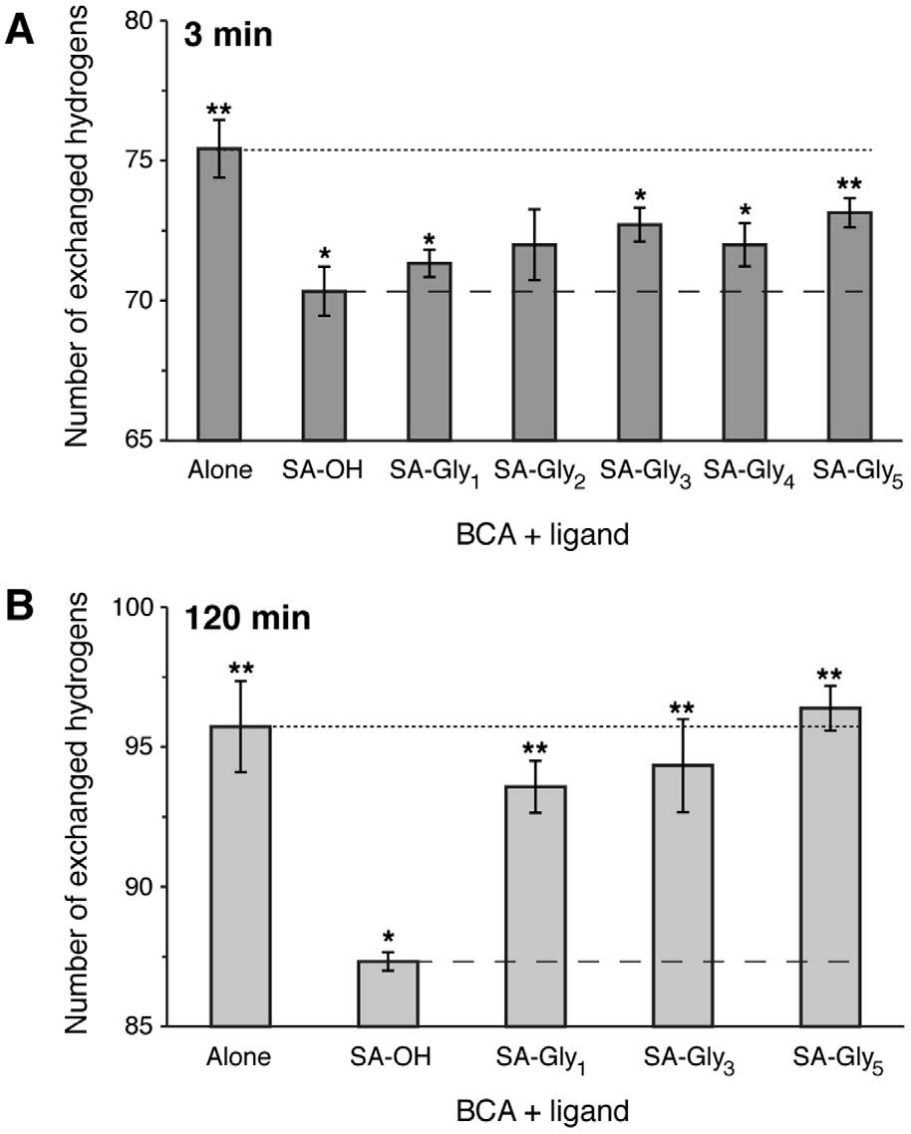
Number of hydrogens in BCA that exchange with deuterium after treatment with or without SA-Gly_n_ ligand. Exchange studies were conducted after incubation in D_2_O for 3 min (A) or 120 min (B), and in both cases reveal a slight trend towards more exchanged protons with increasing length (n) of the SA-Gly_n_ ligand. Levels of statistical significance (Student’s *t*-test, *p*<0.05) are indicated with an ‘*’ for significance over BCA alone (denoted with dotted lines) and ‘**’ for significance over BCA treated with *p*-carboxybenzensulfonamide, SA-OH (denoted with dashed lines). Error bars represent standard errors of the mean of at least five measurements.

Previous measurements of H/D exchange of BCA have shown that BCA exchanges by a predominantly EX2 mechanism [49]. Consequently, the fastest exchanging amides are likely exposed to solvent, or not engaging in hydrogen bonds, whereas the slower exchanging amide hydrogens are buried, hydrogen bonded, or electrostatically shielded from attack by hydroxide (the primary catalyst of amide hydrogen exchange at pH >4 [49], [50], [52]. Thus, we initially used a short incubation time of 3 minutes in D_2_O in order to examine the influence of the ligands on the rates of exchange of the fastest exchanging BCA amide hydrogens. The increase in the mass of BCA, with or without ligand, after incubation in D_2_O relative to the mass of BCA without ligand in H_2_O reveals the number of exchanged hydrogens (i.e., deuterons incorporated) in the protein (Figure 5A). In the absence of ligand, BCA exchanged ∼75 hydrogens after 3 min; a value in good agreement with literature values [49]. The binding of all of the ligands to BCA resulted in a trend to fewer exchanged BCA hydrogens relative to the case when BCA alone was incubated in D_2_O; this decrease was statistically significant (*p*<0.05) for SA-OH, SA-Gly_1_, SA-Gly_3_, and SA-Gly_4_ and at the edge of significance (*p* = 0.06) for SA-Gly_2_ and SA-Gly_5_. This reduction in exchanged hydrogens could be due to better packing of the BCA/ligand complex than of BCA alone, and/or to electrostatic or steric repulsion of the ligand with deuteroxide (OD^-^) that mediates hydrogen exchange at this pH [53]. More importantly, there is a slight trend towards more exchanged BCA hydrogens with increasing chain length from SA-OH to SA-Gly_5_, an observation that is aligned with our expectations for Gly chain-induced protein destabilization. We conducted statistical tests (unpaired Student’s t-test) to determine whether the small variations were statistically significant: BCA incubated with SA-Gly_5_ exchanged more hydrogens than that incubated with SA-OH and SA-Gly_1_ (*p* = 0.02). Although the other differences across the series were not statistically significant (*p*>0.05), the trend of increasing numbers of exchanged hydrogens with chain length of the incubated ligand provides qualitative support for a model based on increasing protein mobility induced by the interaction of the Gly chain of the ligand with BCA.

In order to determine whether the differences might also involve slow-exchanging hydrogens of BCA–amide hydrogens that are at least partially buried from solvent, engaged in strong hydrogen bonding, or electrostatically shielded from deuteroxide–we conducted H/D exchange studies of BCA after a 120-min incubation with D_2_O alone or in the presence of SA-OH, SA-Gly_1_, SA-Gly_3_, or SA-Gly_5_ (Figure 5B). Under these conditions, BCA exchanged ∼96 hydrogens, a value in good agreement with a previous report [49]. BCA incubated with SA-OH exhibited a large and statistically significant reduction in the number of exchanged hydrogens relative to BCA alone (Figure 5B); this reduction was significantly larger than that observed after 3 min (Figure 5A). These observations suggests that the binding of SA-OH results in a greater effect on the exchange of slow-exchanging BCA hydrogens than on the exchange of fast-exchanging hydrogens (probed by the 3 min time point), and are consistent with a model involving better internal packing of BCA when in complex with ligand than when alone (although a model involving steric or electrostatic repulsion of the ligand itself on the exchanging deuteroxide molecule cannot be rigorously eliminated). BCA incubated with the SA-Gly_n_ ligands appeared to exchange slightly fewer hydrogens than BCA alone, although the differences were no longer statistically significant (*p*>0.05); these small differences were qualitatively different from the case at 3 min where BCA in complex with the SA-Gly_n_ ligands exchanged far fewer hydrogens than BCA alone (Figure 5A). This observation implies that some BCA hydrogens (most likely slow-exchanging hydrogens) undergo faster exchange when BCA is in complex with ligand than when it is free. The most likely model for the observed increase in the rate of exchange involves an increase in internal mobility of BCA. Taken together with the low exchange of hydrogens for BCA/SA-OH, which can be seen as the intrinsic effect of the benzenesulfonamide moiety itself, these data imply that a model involving Gly chain-induced destabilization of the structure of BCA is plausible. In addition, there is a trend towards an increase in exchange of BCA hydrogens with chain length of the ligand, with the difference between SA-Gly_1_ and SA-Gly_5_ being statistically significant (*p* = 0.037; Figure 5B).

Overall, the H/D exchange studies suggest that a model in which the Gly chain of the BCA-bound ligand destabilizes internal amides within BCA (i.e., BCA backbone), with this destabilization scaling with the length of the Gly chain, is plausible. Such destabilization should be reflected in a favorable contribution to entropy, and an unfavorable contribution to enthalpy, of binding of SA-Gly_n_ ligands with increasing chain length (n), as a result of fewer well-ordered hydrogen bonds within the protein. This effect would be expected even if the chain itself had negligible interactions (either favorable or unfavorable) with the protein itself. Thus, this model can explain how subunits of the Gly chain that do not appear to make ordered contacts with the protein from X-ray crystallographic analysis (subunits more than three from the benzene ring) [20] might still influence the thermodynamics of binding. This model is consistent with the concept of binding with “negative cooperativity” described by Williams and co-workers [3], in which the mobility at a protein-ligand interface increases when additional interactions are introduced due to the interactions being mutually incompatible.

Importantly, our model involving BCA *backbone* dynamics might be complementary to the simulation results of Homans and co-workers (which suggested conformational mobility of *side chains* of active-site residues), but on the basis of our data we cannot comment on the presence or absence of side-chain conformational mobility.

### Solvation is Likely not Playing a Role in the Underlying Thermodynamics

Previously we had ruled out a model based on increasing hydrophobic contacts between the chain of the ligand and BCA, with chain length (n) because the change in specific heat (Δ*C*_p_)–often referred to as the “signpost” of the hydrophobic effect [22]–[25]–did not vary across the series [18]. While recent reports have suggested that solvent can be implicated in protein-ligand interactions with widely varying thermodynamic profiles (from enthalpically driven to entropically driven), all of these hydrophobic interactions have involved large and significant variations of Δ*C*_p_ across the series of modified ligands [54]–[56]. Given the absence of such a trend in Δ*C*_p_ for the SA-Gly_n_ ligands, a solvation-driven model would seem to be incompatible with the data.

### Conclusions

We have used a combination of biophysical studies (NMR and hydrogen/deuterium exchange) to determine the origin of the perplexing trend of increasingly less unfavorable entropy, and increasingly less favorable enthalpy, in the association of a model protein (bovine carbonic anhydrase II; BCA) with a series of *p*-substituted benzenesulfonamides with oligoglycine chains of variable length (1–5 subunits). Our results demonstrate that the mobility of the ligand in the BCA/ligand complex does not increase with the chain length of the ligand, a result that is consistent with a previous report [26]. Further, our results have revealed that the first Gly subunit (the one closest to the benzene ring) binds without being affected by more distal subunits, and suggest that the same effect holds for the other subunits. We have demonstrated that ligand aggregation, while present in many cases, does not occur in this series of ligands and cannot explain the data. Hydrogen/deuterium exchange studies revealed that, while the benzenesulfonamide moiety itself could plausibly be stabilizing BCA (i.e., reducing its mobility), the Gly chain could be destabilizing it in a manner that scales with the chain length. We, thus, believe that increasing protein mobility with increasing chain length is the origin of the thermodynamic profile of these ligands with the less unfavorable entropy resulting from the increased protein mobility and the less favorable enthalpy from the fewer ordered internal hydrogen bonds, resulting in enthalpy/entropy compensation across the series. To the best of our knowledge, our results represent the first experimental demonstration that the structural dynamics and mobility of BCA change significantly upon the binding of ligands.

We previously reported that benzenesulfonamides with oligo(ethylene glycol) and oligosarcosine chains had similar thermodynamic profiles as the SA-Gly ligands [18]. Given that a model based on an increase in protein mobility with chain length does not require direct interactions between protein and the chain, only that the protein responds to the presence of the chain in the same way, it is plausible that the model might hold for these ligands. Experimental studies would be necessary to verify this hypothesis.

Fragment-based drug discovery efforts, and those based on multivalency, require an understanding of the effect of linking of two or more fragments that bind to adjacent sites of a protein [57]–[62]. While the important role of the linker is well accepted [59]–[62], an equally important consideration is the influence of binding of the fragments on one another [62], [63]. As a simplifying approach to this challenge, a common strategy is to utilize the concept of additivity by assuming that the thermodynamics of binding of a ligand to a protein are equal to the sum of the thermodynamics of binding of the individual components of the ligand (with an appropriate entropic benefit of linking the different components together) [2], [62]–[65]. Challenges to additivity have often focused on the ligand in the protein-ligand complex, invoking arguments of strain on the ligand induced by its inability to bind in an optimal orientation to the multiple sites [3], [62]. Our results suggest that another challenge is in the context of the protein: the Gly subunits of the ligand appeared to bind without being influenced by more distal subunits (in the sense of their mobilities) but their binding resulted in a destabilization of the protein itself. Thus, our work suggests the need for models of protein-ligand binding (e.g., computational approaches) to take into account the role of protein dynamics and mobility [13], [14], even in cases when it would seem not to be relevant.

## Materials and Methods

### General Methods

Chemicals were purchased from Aldrich, Fluka, TCI, Bachem, or ChemImpex at the highest grade available (≥95%), and used as received unless otherwise noted. Bovine carbonic anhydrase II (pI 5.9) was obtained from Sigma. NMR experiments were carried out on a Bruker Avance III 500 MHz or a Bruker Avance-600 MHz. Electrospray ionization-mass spectrometry for hydrogen/deuterium exchanges studies were conducted on a Thermo-Finnigan LTQ Ion Trap ESI mass spectrometer.

### General Procedures for the Synthesis of SA-Gly_n_ Ligands

SA-Gly_1_ was synthesized as previously described [19], but with the substitution of glycine-^15^N (Aldrich; 98 atom % ^15^N) for glycine. Purity of ≥95% was determined by HPLC for all synthesized compounds, and all compounds had masses and ^1^H spectra consistent with their structures. SA-Gly_n_ ligands (n = 2–5) were synthesized using the in-situ neutralization protocol for t-Boc chemistry [66] using *N*-Boc-protected glycine [67] on Boc-Gly-PAM resin (purchased from Chem-Impex International, Inc.). Boc-Gly-OH was used for most steps, with Boc-Gly-OH-^15^N (synthesized from Gly-^15^N) used for the penultimate coupling step, and *p*-carboxybenzenesulfonamide used for the final coupling step. Peptide syntheses were conducted on the 0.05 mmol scale with 0.20 mmol amino acid, 0.18 mmol of HBTU, and 0.30 mmol of diisopropyl ethylamine used for each coupling. Sulfonamide-conjugated peptides were cleaved by HF/anisole (90%/10%) at 0°C for 2 h and precipitated with cold ether. Crude molecules were purified by RP-HPLC (Hitachi D-7000) on a C18 column (Vydac, 10 × 250 mm, 10 µm) using a linear gradient of 99% water/1% acetonitrile with 0.1% TFA (solvent *A*) followed by 90% acetonitrile/10% water containing 0.07% TFA (solvent *B*), at a flow rate of 8 mL min^−1^ (UV detection at 254 nm). Purified molecules were characterized by ^1^H NMR spectroscopy (Bruker Avance III 500 MHz), high-resolution electrospray ionization mass spectra (HRMS; Bruker Maxis Impact LC-q-TOF Mass Spectrometer) in the negative-ion mode, and analytical RP-HPLC on a C18 column (Vydac C18RP, 4.6 × 250 mm, 7 µm) at a flow rate of 1.5 mL min^−1^ (UV detection at 254 nm).

### *p*-H_2_NSO_2_C_6_H_4_CO^15^NHCH_2_CO_2_H (SA-Gly_1_)

HPLC *t*_R_ 9.50 min (linear gradient, 0–15% *B*, 20 min); ^1^H NMR (500 MHz, D_2_O) δ 8.03 (d, *J = *8.5 Hz, 2H), 7.99 (d, *J = *9.0 Hz, 2H), 4.10 (s, 2H); HRMS: 258.0202. Calcd for C_9_H_9_N^15^NO_5_S^−^ (M−H^−^): 258.0208.

### *p*-H_2_NSO_2_C_6_H_4_CO^15^NHCH_2_CONHCH_2_CO_2_H (SA-Gly_2_)

HPLC *t*_R_ 7.87 min (linear gradient, 0–15% *B*, 20 min); ^1^H NMR (500 MHz, D_2_O) δ 8.09 (d, *J = *8.5 Hz, 2H), 7.99 (d, *J = *8.5 Hz, 2H), 4.24 (s, 2H), 3.93 (s, 2H); HRMS: 315.0421. Calcd for C_11_H_12_N_2_^15^NO_6_S^−^ (M−H^−^): 315.0423.

### *p*-H_2_NSO_2_C_6_H_4_CO^15^NHCH_2_CO(NHCH_2_CO)_2_OH (SA-Gly_3_)

HPLC *t*_R_ 8.22 min (linear gradient, 0–15% *B*, 20 min); ^1^H NMR (500 MHz, D_2_O) δ 8.03 (d, *J = *8.0 Hz, 2H), 8.00 (d, *J = *9.0 Hz, 2H), 4.18 (s, 2H), 4.01 (s, 2H), 3.89 (s, 2H); HRMS: 372.0634. Calcd for C_13_H_15_N_3_^15^NO_7_S^−^ (M−H^−^): 372.0637.

### *p*-H_2_NSO_2_C_6_H_4_CO^15^NHCH_2_CO(NHCH_2_CO)_3_OH (SA-Gly_4_)

HPLC *t*_R_ 8.50 min (linear gradient, 0–15% *B*, 20 min); ^1^H NMR (500 MHz, D_2_O) δ 8.04 (d, *J = *8.5 Hz, 2H), 8.00 (d, *J = *9.0 Hz, 2H), 4.20 (s, 2H), 4.03 (s, 2H), 3.99 (s, 2H), 3.74 (s, 2H); HRMS: 429.0845. Calcd for C_15_H_18_N_4_^15^NO_8_S^−^ (M−H^−^): 429.0852.

### *p*-H_2_NSO_2_C_6_H_4_CO^15^NHCH_2_CO(NHCH_2_CO)_4_OH (SA-Gly_5_)

HPLC *t*_R_ 9.07 min (linear gradient, 0–15% *B*, 20 min); ^1^H NMR (500 MHz, D_2_O) δ 8.03 (d, *J = *8.5 Hz, 2H), 8.00 (d, *J = *8.5 Hz, 2H), 4.19 (s, 2H), 4.04 (s, 2H), 4.02 (s, 2H), 3.95 (s, 2H), 3.73 (s, 2H); HRMS: 486.1065. Calcd for C_17_H_21_N_5_^15^NO_9_S^−^ (M−H^−^): 486.1067.

### NMR Measurements of Relaxation Parameters

Relaxation times of free SA-Gly_n_ ligands were measured on samples of ligand (500 µM; concentration of D_2_O stock solutions determined by ^1^H NMR spectroscopy) [18] in 20 mM sodium phosphate buffer pH 6.8, with 10 µM sodium azide in 90% H_2_O/10% D_2_O on a Bruker Avance-600 MHz spectrometer at 298 K using the standard Bruker pulse sequences from Farrow et al. [36]: invit1etf3gpsi for *T*_1_ and invit2etf3gpsi for *T*_2_. Values of ^15^N *T*_1_ were obtained from HSQC spectra recorded with delay times of 0.05, 0.25, 0.5, 1, 2, 3, 4, 6, and 10 s, and values of ^15^N *T*_2_ from spectra with delay times of 0.016, 0.096, 0.256, 0.512, 0.736, 0.992, 1.504, 2.000, 2.992, 5.008 s. Peak intensities from the HSQC spectra were fit to a two-parameter exponential decay function using non-linear least-squares fitting to obtain estimates for relaxation times and uncertainties. Duplicate spectra were recorded at 2–3 of the delay times and demonstrated variations of <10% in peak intensities.

Relaxation times (*T*_1_ and *T*_2_) of SA-Gly_n_ ligands in complex with BCA were measured as described above but samples also contained BCA (600 µM; concentration determined by UV spectroscopy with ε_280_ = 55,300 M^−1^ cm^−1^) [21], and the pH was 7.5. Values of *T*_1ρ_ and ^15^N-^1^H steady-state NOE were estimated using the pulse sequences hsqctretf3gpsi [68] and invinoef3gpsi [36], respectively. Values of ^15^N *T*_1_ were obtained from HSQC spectra recorded with delay times of 10.9, 54.3, 108.6, 217.3, 380.2, 543.2, 760.5, 997.8, 1303.7, and 1629.6 ms, and values of ^15^N *T*_2_ and *T*_1ρ_ from spectra with delay times of 16, 32, 48, 64, 96, 128, 160, 192, 224, and 256 ms. Peak intensities from the HSQC spectra were fit to a two-parameter exponential decay function using non-linear least-squares fitting to obtain estimates for relaxation times and uncertainties. Estimates of ^15^N-^1^H steady-state NOE were obtained from the ratio of peak intensities from HSQC spectra recorded with ^1^H saturation to those recorded without saturation, and corrected to account for the incomplete magnetization recovery during the relaxation delay period of 3 s [69], [70]. Uncertainties were estimated from the range in values of NOE from replicate (2–3) experiments.

### Model-free Analysis of NMR Data

The extended model-free order parameters for all five models [31]–[33] for the SA-Gly_n_ ligands in complex with BCA were obtained using the software program Modelfree version 4.20 and the experimental NMR relaxation parameters with associated uncertainties. The overall rotational correlation time for BCA was fixed at 11.5 ns to facilitate comparison with the report of Homans and co-workers [26]. Uncertainties in the order parameters were estimated from 500 Monte Carlo simulations. Modelfree-derived values of *χ* ^2^ were used to calculate values of AIC for the ligands in each model with eq 3 [37], [38]:
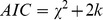
(3)where k is the number of model-free parameters allowed to vary in the fitted model.

### Diffusion-ordered NMR Spectroscopy (DOSY)

DOSY experiments were performed on samples of free SA-Gly_n_ ligands (prepared as for the NMR relaxation studies with the addition of 4,4-dimethyl-4-silapentane-1-sulfonic acid, DSS, as internal standard) at 300 K on a Bruker Avance III 500 MHz spectrometer using a pulsed field gradient stimulated echo sequence with 3-9-19 WATERGATE suppression of the H_2_O signal (Bruker standard pulse sequence: stebpgp1s19). The diffusion time (Δ) and gradient length (*δ*) were held constant at 100 ms and 2.5 ms, respectively; this choice of parameters achieved the desired 90–95% decrease in resonance intensity at 95% of the total gradient strength (53.5 G cm^−1^) for all of the samples. The gradient strength was linearly incremented in 16 steps from 2% to 95% of the total gradient strength. The ^1^H spectra were analyzed using the standard 2D DOSY processing protocol in the Topspin 2.1 (Bruker) software package to obtain the DOSY spectra (Figure S2) from which diffusion coefficients of the SA-Gly_n_ ligands were extracted.

### Hydrogen/deuterium Exchange Studies

Amide hydrogen/deuterium exchange studies were performed as previously described [49], using a Thermo-Finnigan LTQ Ion Trap ESI-MS. Briefly, solutions of BCA were incubated with each ligand and concentrated to approximately 20 mg mL^−1^ (100 mM phosphate buffer, pH 7.4). Solutions were then diluted 1∶10 in isotopically pure D_2_O (99.9% D). At 3 min or 120 min after dilution into D_2_O, aliquots (7.5 µL) were removed and H/D exchange was quenched by immediate freezing in N_2_ (l). For mass spectrometric analysis, samples were immediately thawed and acid quenched by adding 142.5 µL of cold formic acid (0.3%) to each tube that contained the frozen aliquot (7.5 µL). The addition of cold formic acid to this small volume of frozen protein solution resulted in instantaneous thawing. The sample was then immediately loaded onto a Rheodyne injector that was connected to a short desalting column (Michrom Bioresources Inc.) immersed in ice. The injector was situated less than 12 inches from the ion source of the ESI-MS. After loading each sample onto the desalting column, the column was washed with 1.5 mL of formic acid via injection by syringe. The protein was then eluted from the desalting column and analyzed with ESI-MS by elution with 60% acetonitrile/H_2_O, 0.3% formic acid. In order to estimate the amount of back exchange that occurred for the BCA protein/ligand complex during quenching and ESI-MS analysis, an aliquot from each BCA/D_2_O/ligand solution was removed and perdeuterated by thermally unfolding each aliquot of protein at 80°C. Each perdeuterated protein was then frozen, thawed, and analyzed in the exact same manner as for the native protein. The measured amount of back-exchange–the difference between the experimentally observed mass of perdeuterated BCA and its theoretical mass (calculated assuming that 90% of the available amide hydrogens of BCA have been exchanged with deuterium [49])–was 35%.

## Supporting Information

Figure S1 **Ratio of** ***T*** **_1ρ_ to** ***T*** **_2_ as a function of chain length (n) for SA-Gly_n_ ligands complexed to BCA.** The ratios for all ligands are unity within the uncertainties of the data. Error bars represent uncertainties propagated from uncertainties in the individual relaxation parameters.
(TIF)

Figure S2 **^1^H-detected Diffusion-Ordered NMR SpectroscopY (DOSY) spectra of SA-Gly_n_ ligands.** Samples were in 20 mM sodium phosphate pH 6.8 in 90% H_2_O : 10% D_2_O: A) n = 1, B) n = 2, C) n = 3, D) n = 4, E) n = 5. The aryl protons of SA-Gly_n_ appear at ∼8 ppm and were used to estimate diffusion coefficients of the ligands; alpha protons appear in the range 3.6–4.4 ppm. The DSS internal standard was referenced to 0 ppm. The water peak appears at ∼4.7 ppm. Unassigned contaminants in the phosphate buffer are labeled with ‘*’; these contaminants were not present in NMR spectra of the pure compounds.
(TIF)
