# Supplementary figures and images for: Ligand-Induced Protein Mobility in Complexes of Carbonic Anhydrase II and Benzenesulfonamides with Oligoglycine Chains
Source: PLoS One. 2013 Mar 5;8(3):e57629. doi: 10.1371/journal.pone.0057629 (PMC3589393; doi:10.1371/journal.pone.0057629)

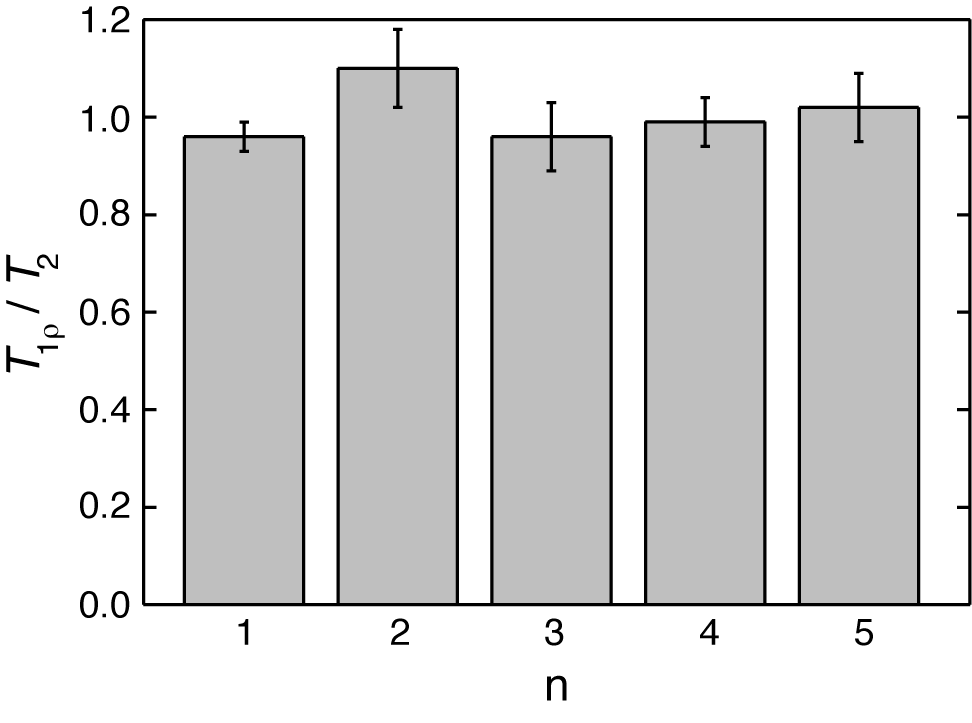

Supplement: Figure S1 — Ratio of T 1ρ to T 2 as a function of chain length (n) for SA-Glyn ligands complexed to BCA. The ratios for all ligands are unity within the uncertainties of the data. Error bars represent uncertainties propagated from uncertainties in the individual relaxation parameters. (TIF) [file pone.0057629.s001.tif]

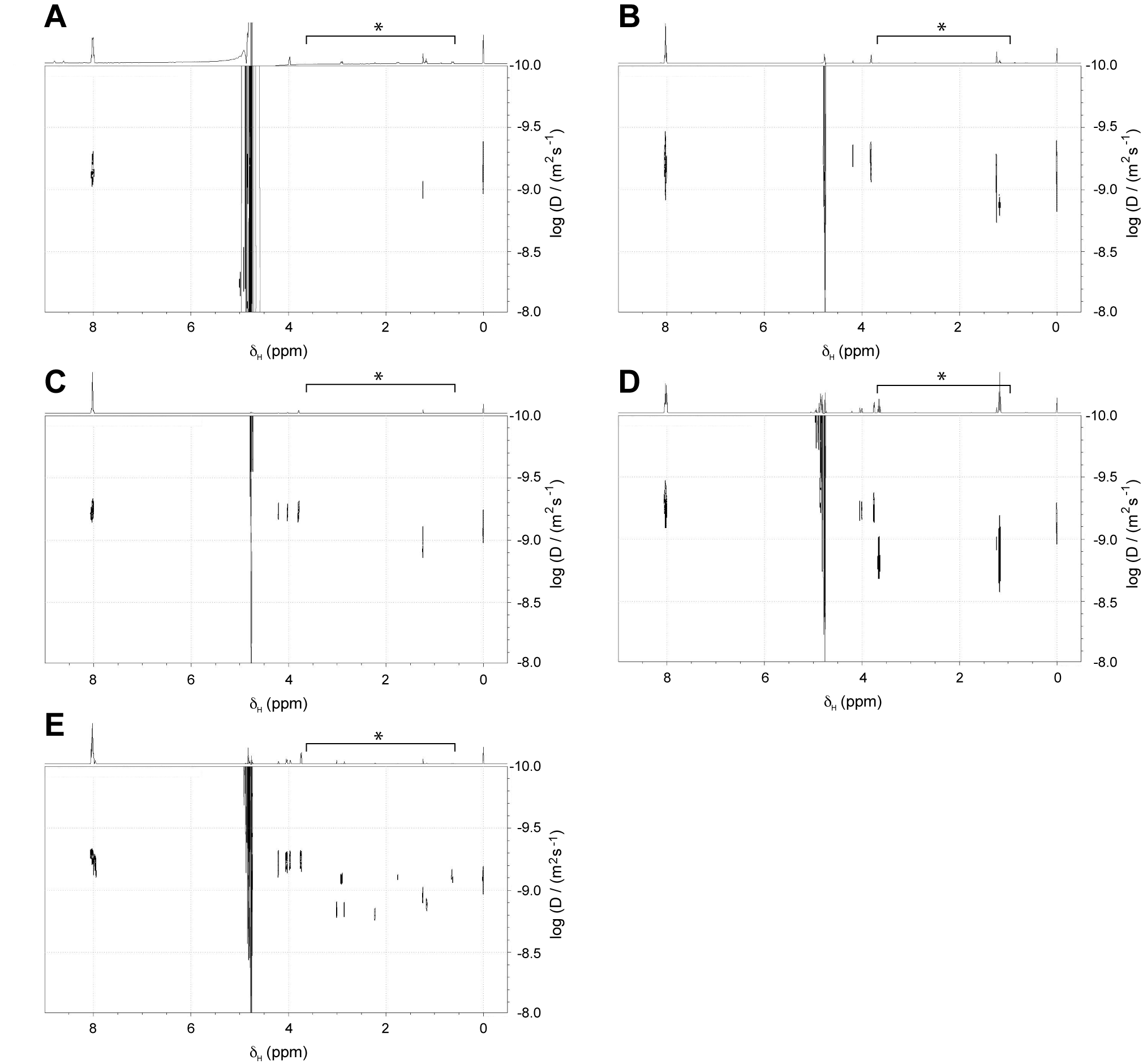

Supplement: Figure S2 — 1H-detected Diffusion-Ordered NMR SpectroscopY (DOSY) spectra of SA-Glyn ligands. Samples were in 20 mM sodium phosphate pH 6.8 in 90% H2O : 10% D2O: A) n = 1, B) n = 2, C) n = 3, D) n = 4, E) n = 5. The aryl protons of SA-Glyn appear at ∼8 ppm and were used to estimate diffusion coefficients of the ligands; alpha protons appear in the range 3.6–4.4 ppm. The DSS internal standard was referenced to 0 ppm. The water peak appears at ∼4.7 ppm. Unassigned contaminants in the phosphate buffer are labeled with ‘*’; these contaminants were not present in NMR spectra of the pure compounds. (TIF) [file pone.0057629.s002.tif]
